# Congenital Malformations in River Buffalo (*Bubalus bubalis*)

**DOI:** 10.3390/ani7020009

**Published:** 2017-02-10

**Authors:** Sara Albarella, Francesca Ciotola, Emanuele D’Anza, Angelo Coletta, Luigi Zicarelli, Vincenzo Peretti

**Affiliations:** 1 Department of Veterinary Medicine and Animal Production, University of Naples Federico II, via Delpino 1, Naples 80137, Italy; sara.albarella@unina.it (S.A.); emanuele.danza@unina.it (E.D.A.); zicarell@unina.it (L.Z.) vincenzo.peretti@unina.it (V.P.); 2 Associazione Nazionale Allevatori Specie Bufalina–ANASB, Caserta 81100, Italy; espertidirazza@anasb.it

**Keywords:** congenital malformation, river buffalo, disorders of sexual development, genetic musculoskeletal defects

## Abstract

**Simple Summary:**

Congenital malformations (due to genetic causes) represent a hidden danger for animal production, above all when genetic selection is undertaken for production improvements. These malformations are responsible for economic losses either because they reduce the productivity of the farm, or because their spread in the population would decrease the total productivity of that species/breed. River buffalo is a species of increasing interest all over the world for its production abilities, as proved by the buffalo genome project and the genetic selection plans that are currently performed in different countries. The aim of this review is to provide a general view of different models of congenital malformations in buffalo and their world distribution. This would be useful either for those who performed buffalo genetic selection or for researchers in genetic diseases, which would be an advantage to their studies with respect to the knowledge of gene mutations and interactions in this species.

**Abstract:**

The world buffalo population is about 168 million, and it is still growing, in India, China, Brazil, and Italy. In these countries, buffalo genetic breeding programs have been performed for many decades. The occurrence of congenital malformations has caused a slowing of the genetic progress and economic loss for the breeders, due to the death of animals, or damage to their reproductive ability or failing of milk production. Moreover, they cause animal welfare reduction because they can imply foetal dystocia and because the affected animals have a reduced fitness with little chances of survival. This review depicts, in the river buffalo (*Bubalus bubalis*) world population, the present status of the congenital malformations, due to genetic causes, to identify their frequency and distribution in order to develop genetic breeding plans able to improve the productive and reproductive performance, and avoid the spreading of detrimental gene variants. Congenital malformations most frequently reported in literature or signaled by breeders to the Department of Veterinary Medicine and Animal Production of the University Federico II (Naples, Italy) in river buffalo are: musculoskeletal defects (transverse hemimelia, arthrogryposis, umbilical hernia) and disorders of sexual development. In conclusion this review put in evidence that river buffalo have a great variety of malformations due to genetic causes, and TH and omphalocele are the most frequent and that several cases are still not reported, leading to an underestimation of the real weight of genetic diseases in this species.

## 1. Introduction

The world’s water buffalo population is about 168 million: 95% are in Asia (India, Pakistan, and China), 2% in Africa (Egypt), 2% in South America and the Caribbean, 1% in Europe and Oceania [1] Buffalo bred for milk production belong to the River type, like Murrah and Jaffarabadi (both bred in Brazil, also for meat production), Nili Ravi, Mediterranean River Buffalo, and Mediterranean Italian River Buffalo (MIRB). Swamp buffaloes, bred in South East Asia, are draft animals used extensively in paddy field farming.

Breeding programs are carried out in many countries, such as China, India, and Brazil; however, Italy represents the international point of reference for buffalo breeding as a result of the innovations brought in management, dairy technologies, cheese manufacturing, feeding, and reproduction. Despite the aim of breeding plans being to obtain healthy animals with good morphology and high production, it is not uncommon for animals to be born showing congenital defects due to gene mutations inherited from the parents or caused by new mutations or changes to the DNA. The occurrence of congenital malformations, above all when due to gene variants passed down by parents, in livestock production, causes the slowing of genetic progress, economic loss for the breeders due to the death of animals, or damage to their reproductive and productive ability, such as milk production. Moreover, congenital malformations imply a reduction of animal welfare directly in the affected animals because they have a reduced fitness and a lower adaptability to farming conditions and indirectly because can provoke foetal dystocia. To study the genetic bases of these diseases, and to find strategies to eradicate them in livestock populations is, for breeders, an issue to avoid economic losses [2].

With respect to the worldwide presence of congenital malformations in River buffalo, there are few papers, and a systematic report is still lacking. Purohit et al. [3] reported that 1.78% of dystocic deliveries from fetal causes are due to fetal malformations. In Brazil, Marcolongo-Pereira et al. [4] observed in the Murrah breed the occurrence of congenital defects in 7.54% of buffalo specimens necroscopically examined between 1978 and 2009. The most frequent congenital defects were arthrogryposis (seven animals), myotonia (29 animals), and mechano-bullous genodermatoses (four animals). According to Damè et al. [5] all of these defects were due to the high level of consanguinity of Brazilian buffaloes. In MIRB, transverse hemimelia [6] and sexual developmental abnormalities [7] were mainly reported (114 and six cases, respectively). According to the World Health Organization, congenital diseases are structural or functional anomalies, including metabolic disorders, which are present at the time of birth due to different etiology causes, such as genetic and environmental factors and infections [8]. 

This paper aims to provides an overview of the congenital malformations reported in river buffalo (*Bubalus bubalis*) in the literature or that have been signaled (and still unpublished) to the Department of Veterinary Medicine and Animal Production of University of Naples Federico II by ANASB and veterinarians, most likely due to genetic causes, according to available information, because of their increasing impact in the world livestock production chain. Moreover, the completion of buffalo genome sequencing and annotation will allow the identification of gene mutations underlying these malformations. 

The congenital malformations observed can be classified as: musculoskeletal defects, gastrointestinal defects, craniofacial abnormalities, disorders of sexual development, and other malformations (Table 1).

## 2. Musculoskeletal Defects

### 2.1. Transverse Hemimelia

Transverse hemimelia (TH) is a congenital malformation characterized by total or partial lack of distal limb structures. It is also known as congenital amputation, since the malformed limb is similar to a stump normally developed proximally and distally, but cut off at different points. 

In river buffalo, and particularly in MIRB, this is the most frequent congenital malformation diagnosed previously and, similar to other species, all of the cutaneous annexes are present so that in the terminal part of the stump, the sketch of hooves are visible. It was reported for the first time in some farms located in the province of Salerno [9], and then in different farms of Southern Italy [6,10]. 

At birth buffalo calves with TH are alive and lively, the only symptom is difficulty in moving due to the malformed limb or limbs. At necropsy they show no macroscopic lesions incompatible with life and their karyotype is normal. In Table 2 the cases analyzed in our lab are reported, classified according to phenotypic characteristics; the most frequent lesion is TH of the hock of one hind limb. Anamnestic data and examinations performed on malformed calves and their parents permitted us to suppose the action of one or more autosomal genes at the base of the malformation. Thanks to a genomic sequencing project the genome of 11 affected buffaloes was sequenced and compared with that of 14 healthy controls revealing that variants of *SMARCA4* and *WNT7A* genes are the main drivers for this type of TH malformation. Moreover, the accumulation of homozygous mutations in modifier genes appears to impact the severity of the TH phenotype, resulting in animals that vary from the lack of a single transverse bone in one limb to the complete lack of both hindlimbs with malformation of one forelimb [11], and probably to the lack of all four limbs. Another case that can be classified as TH has been reported by Jethva et al. [12], that observed an Indian Mehsana buffalo male with no hind limbs. More recently, a case of TH has been reported in a buffalo fetus with multiple craniofacial defects [13]. In both cases a genetic base is only supposed, but not supported, by other data.

### 2.2. Arthrogryposis

Arthrogryposis is a congenital disease characterized by non-progressive joint contractures that can affect upper or lower limbs and/or the vertebral column, leading to various degrees of flexion or extension limitations, evident at birth. It is not a specific diagnosis, but rather a clinical finding of congenital contractures causing severe mobility difficulties in affected individuals. Arthrogryposis has been reported in livestock and pets and is often associated with muscle atrophy or other malformations. It has more than one etiological factor, including physical limitation of in utero movement causing fetal akinesia/hypokinesia syndrome, maternal illness, intrauterine viral infection (Schmallenberg virus, Akabane virus, or Aino virus), toxin exposure, and genetic disorders affecting the fetus [14]. In Horse, this malformation has been associated with trisomy [15] while in sheep a candidate region for arthrogryposis has been identified on the proximal end of OAR5 [16]. In cattle arthrogryposis multiplex congenita has been associated with a genomic deletion encompassing *ISG15*, *HES4*, and *AGRN* genes in the American Angus breed [17]; and in Red Dairy cattle it is associated with a deletion in the first exon of *CHRNB1* [18]; furthermore, Iannuzzi et al., in a Piedmont calf affected by arthrogryposis, found the duplication of the *SMN* gene on BTA20q13.1 [19]. In Southern Brazil Schild et al. [20] found a Murrah buffalo herd in which the disease was transmitted as an autosomal recessive disorder, moreover, in India, Saini et al. [21] reported a case of a Murrah buffalo male fetus born dead with flexions and multiple articular rigidity of joints of all four limbs. Finally, Correale and Consalvo [9] observed, in MIRB, a case of a calf born with an abnormal and permanent ankylosis of limb joints. In the cases described by Saini et al., and Correale and Consalvo, the involvement of all four limbs let us assume gene abnormalities are responsible for the malformation.

### 2.3. Other Musculoskeletal Defects

A variety of other musculoskeletal malformations have been reported in river buffalo. Mehmood et al. [22] described a case of congenital perosomus elumbis (PE) in a Nili Ravi buffalo characterized by small vertebral column, under-developed pelvis, and deformed hind limbs. PE has also been reported in other animal species, such as cattle, mainly in the Holstein Friesian breed, sheep, pigs, dogs, and horses [23]. The etiology of PE is still not known, but mutations within the homeobox (Hox) gene family are believed the principal causes of this malformation because they are involved in fundamental ontogenetic processes [24,25].

Kumar et al. [26] observed a case of *campylorrhacchis contorta* in a calf born from a primiparous Indian buffalo; the fetus was under-weighed and showed brachignathism/parrot mouth, malformation of the pelvis, lumbar, and sacral regions, and ankylosis of all of the limbs with atrophy of musculature. According to the author, this malformation being a congenital defect is most likely caused by a defective gene or a genetic insult.

Another congenital musculoskeletal defect reported in river buffalo is myotonia congenita; a specific inherited disorder of muscle membrane hyperexcitability. It is caused by reduced sarcolemmal chloride conductance due to mutations in *CLCN1*; the gene coding for the main skeletal muscle chloride channel *ClC-1* [27]. It has been also reported in 29 Murrah buffaloes [4], inherited in an autosomal recessive manner and due to an aberrant splicing of *CLCN1* associated to a synonymous SNP in exon-3 (c.396C>T). All animals showed generalized muscular hypertrophy and action myotonia, especially after being startled with difficulties in rising from recumbency and stiffness at the beginning of the movements [28].

Congenital megaesophagus (ME) consists of esophageal dilation and dysmotility that progressively leads to the inability to drive the food/bolus into the stomach. It has been reported in cats [29], dog [30], horses [31], cattle [32], and buffalo [5]. The etiology of ME is still unclear, and GWAS and whole genome sequencing analyses performed in cats and dogs showed that different genes could be related to this condition, like *COLQ* gene in cats [29], and genes located in a region of CFA12 in the German Shepherd dog breed [30]. 

Nine cases of ME have been reported in Murrah buffaloes [5] in which genealogical data permitted the establishment that the condition is inherited. Affected animals showed a short tail, delayed growth, chronic bloat, regurgitation, and aspiration pneumonia. They died at 3–9 months of age and, from an anatomophatological point of view, they showed dilated and obstructed esophagus with flaccid musculature, edema, emphysema, and the presence of liquids in the thoracic cavity.

In addition to the previously described congenital malformations involving limbs, at the Department of Veterinary Medicine and Animal Production of the University Federico II of Naples, in MIRB, we have examined a case of forelimb amelia and a calf with a complex of skeletal malformation involving only limbs characterized by hypoplasia of the radius and ulna, the absence of medial femoral condyle, and tibial agenesis (unpublished data). Embrionic limb development is a complex process under the control of several genes, like *SHH*, *GLI3*, *WNT7A*, *Fgfs*, *BMPs*, *ALX4*, *PRMT5*, and *SMARCA4*; thus, they can all be considered candidate genes for these malformations. Recently, a duplication of exon 2 of the *ALX4* has been observed in Galloway Cattle affected by tibial hemimelia syndrome and assumed to be the candidate causative mutation for this malformation [33].

### 2.4. Umbilical Hernia 

Congenital umbilical hernia (omphalocele) is the protrusion, in a newborn, of the peritoneal sac and, eventually, of other abdominal organs that are covered by skin and subcutaneous tissue through the abdominal wall at the umbilical level [34]. It is due to the failure of the closure of the umbilical ring at birth and its severity varies in relation to the extent of the umbilical defect and the amount of the protruding abdominal organs. In small and large animals, it is a common defect and a genetic component is believed to be involved in this malformation. In pigs it is the most relevant congenital disorder and two gene loci have been associated to this malformation: *SWR1928* on SSC7 and *SW830* on SSC10 [35]. In this species, the *CDC73* gene is assumed to be responsible for the malformation [36]. In cattle this condition is the most frequently noted congenital disorder [37] and it seems to be associated with genes located at the centromeric end of BTA8 [38]. Additionally, this is the most common malformation in river buffalo, but only a few cases are well described in the literature, like in the Pandharpuri buffalo breed in which Amle et al. [39] found a male calf with incomplete closure of umbilicus and reducible intestinal hernia associated to cryptorchidism, and in MIRB, in which Correale and Consalvo [9] found 72 cases with different phenotypic traits. Genetic causes in this species have never been studied and it could be interesting to verify if, in river buffalo, responsible genes are located on the long arm of BBU3 homologue of BTA8 [40].

### 2.5. Schistosoma Reflexum Syndrome

Schistosoma Reflexum or schistosomus reflexus (SR) syndrome is a rare and fatal congenital malformation characterized by exposed abdominal, and sometimes thoracic, viscera [41], failure or incomplete closure of abdominal wall, and marked dorsoflexion and ankylosis of the spine and limbs [42]. Other malformations, like fused ribs and vertebrae, fissure of genitals, and polypodia may also appear. Kumar et al. [43] reported a case of dystocia in a Murrah buffalo due to a fetus with schistosoma reflexum characterized by distinct ventral curvature of the spine, incomplete development of diaphragm attachment and thoracic arc, exposition of abdominal visceral organs, like intestines, omentum, kidney, and liver, and limbs that were touching each other, while the neck and head were in an ankolysed state. In MIRB, Correale and Consalvo [9] reported the birth of two calves with exposition of the abdominal organs and dorsoflexion of the spine; both calvings were at full term pregnancy, but dystocic. In particular, one calf showed a somathopleure dorsal tilt, instead of ventrally, closes on itself, like a bag formed with amnios, a dorsal bag in which limbs were gathered. Moreover, ribs were sternalized on the back, thus resembling a reverse thorax. These cases were diagnosed as SR syndrome. No etiology was supposed in both reports and no genetic analyses has been performed, but it has been reported that an autosomal recessive mode of inheritance for SR exists in pets [44] and in cattle [42].

## 3. Gastrointestinal Defects

### Atresia Ani

Atresia ani is a congenital malformation of the anorectum due to the failure of the urorectal fold to divide the cloaca completely or of the failure of the perforation of the fetal anal membrane that divides the rectus and anus during fetal development. As a consequence, the anal opening is closed and feces are accumulated in the large intestine. This is the most common defect of the lower gastrointestinal tract reported in mammals [45] and, in particular, in cattle, pigs, and sheep [46], while it has been reported few times in horses and donkeys [47]. In pigs a polygenic inheritance, autosomal recessive in some cases and autosomal dominant transmission in others, has been observed [48] and genome analysis allowed for the identification of candidate loci for atresia ani on SSC1, 9, and 15 [49,50], and *GLI2* as a candidate gene [51]. In cattle, several cases of atresia ani are reported [45,52,53,54,55] mainly in Holstein Friesian. In this breed there is some evidence that this condition is inherited in an autosomal recessive manner, but no candidate genes have been found. In MIRB, Correale and Consalvo [9] reported two cases in which the rectum is normally developed but ends in a blind pouch due to the persistence of a complete anal membrane. No data are available about the mode of its inheritance in buffalo or its etiology, but the development of the hindgut is governed by multiple genes in the relevant signaling pathways. Based on the evidence of human genetic studies, murine gene knock-out and teratogenic models, and epidemiological studies, anorectal malformation (ARM) may, most likely, be the result of genetic susceptibility and epigenetic interaction. Genetic factors have been considered important contributing factors in the pathogenesis of ARM [56].

## 4. Craniofacial Abnormalities

### 4.1. Hydrocephalus

Hydrocephalus is a multifactorial, congenital, or acquired disorder characterized by an abnormal accumulation of cerebrospinal fluid (CSF) within the cranial cavity. It is defined as internal when the CFS is accumulated in the ventricles, and external when CFS is accumulated in the subarachnoid space [57]. The increase of CFS is, above all, related to its abnormal reabsorption or defective lymphatic drainage and rarely to its production, and it induces progressive enlargement of the head. Etiology includes: genetic factors, developmental anomalies, intrauterine or prenatal infection, in utero teratogens exposure, dietary deficiencies (vitamin A deficiency in the rabbit), or tumors or bleeding in the brain. Between 1978 and 2009 from 61 autopsies of Murrah buffaloes, Marcolongo-Pereira et al. [4] diagnosed seven cases of hydranencefalia, probably due to hereditary factors, one of which was associated to the condition of albinism. The histopathological examination of this last case revealed the presence of cystic cavities in the thalamus and the substitution of nervous tissue with liquid; moreover, the cortex was thinner. Pandey et al. [58] described a case of hydrocephalus in a male “Bull Dog” calf as shown in Section 4.3. Sharma et al. [59] described the case of dystocia due to hydrocephalic monster as a buffalo calf characterized by an extraordinarily large, football shaped head; on dissection, cranial bones were found markedly thin. Although genetic factors are the most frequent cause of this malformation, the authors have not fully evaluated intra-uterine infections and nutritional factors. Thus, the causes cannot be ruled out in this case. In MIRB Correale and Consalvo [9] have found three cases of hydrocephalus with typical alterations consisting of dilation of cerebral ventricles due to abnormal liquor accumulation and consequent increase of cranium volume. The nervous structures were reduced due to the pressure caused by the increased liquor volume; the calves were born dead or died within a few days after birth. No other malformations are described in those three cases and the possible action of infections and teratogen substances, such as drugs or toxins, is not supposed by the authors. This lets us assume that, in all of the cases, a genetic cause is the most likely cause.

### 4.2. Polycephaly

Polycephaly is a congenital malformation in which an individual with two (dicephaly) or more heads, probably due to partial or total union of two developing embryos or to a partial duplication of a body, or to the anteroposterior compression of the embryonic disk [60]. It is very rare and the affected individual, typically, is either stillborn or dies very soon after birth. It has been reported in different mammals alone, or associated with other malformations, in cattle [61,62,63,64], sheep [65], and goats [66] but, as for other congenital abnormalities, polycephaly is likely to occur more frequently than the veterinary literature would suggest and only in few cases is it possible to hypothesize genetic mutations in the etiology. The most frequent type of polycephalia observed is dicephalia, which is classified in: atlodymus (two complete and separate skull and one neck), iniodymus (two skulls with fusion at the occipital level), and derodymus (two complete and separate skulls with two separate necks [60].

In buffaloes several cases of duplication of the head with different characteristics have been reported: monster with varying degrees of duplication of the face or diprosopus [67] and dicephalus monsters characterized by two full developed heads of similar size fused at the level of atlas [60,68,69,70].

**Figure 1:**
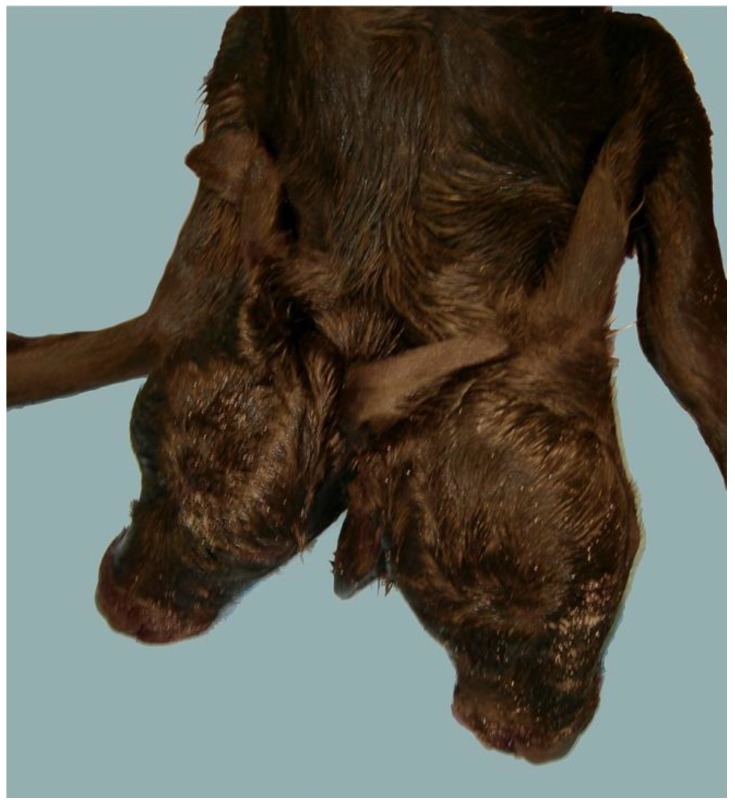
Dicephalic derodymus newborn MIRB calf with complete duplication of cranial structures up to the neck.

In MIRB, we have seen a case of a dicephalic derodymus calf (Figure 1) characterized by complete duplication of cranial structures (two muzzles, four eyes, and four ears) and organs up to the neck. The calf was born dead after a dystocic calving, thus cytogenetic analyses were not performed, while according to genetic analyses performed the calf is not the fusion of two developing embryos (unpublished data). 

### 4.3. Other Craniofacial Malformations

Other congenital craniofacial malformations are very rare in domestic animals and the most frequent reported are brachygnatia, prognathia, micrognathia, clefts of palate, of the face, or of specific facial boneshave. In some cases an inherited condition has been demonstrated [71,72] and sometimes underlying chromosome regions are involved [73], or genetic mutations [74] have been detected. 

**Figure 2:**
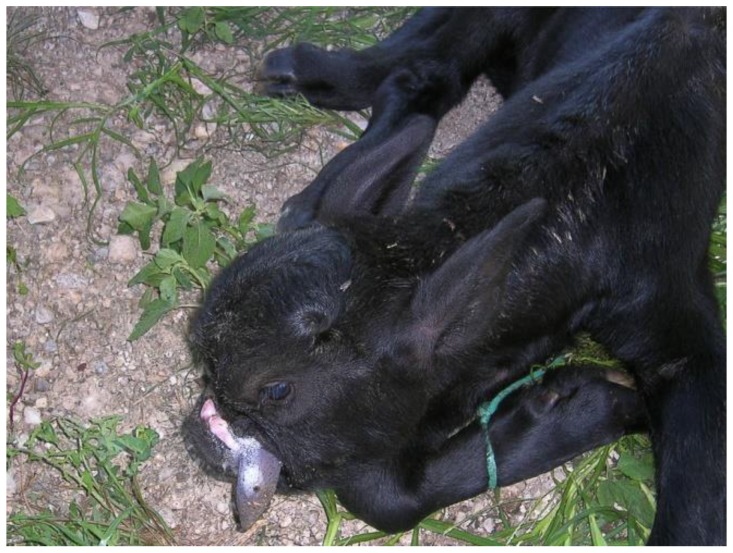
A newborn MIRB calf having underdeveloped incisive, maxillary, and nasal bones with a consequent tongue prolapse and lower jaw deformities.

In India, three cases of “Bull Dog” buffalo calves have been observed; the first one was from a Murrah buffalo fetus of 8.5 months characterized by complete alopecia, broad head, bulging forehead, malocclusions of the jaw, prognathism of the mandible, and pot belly [75]; the second case was a dead male calf from the Surti breed with prognathism, protruding tongue, nose divided by furrows, hydrocephalus, and the whole body covered by hairs [58]. In both cases, thoracic and visceral organs were normal. A third case from a Murrah buffalo has been reported by Singh et al. [76], which was characterized by an enlarged head, bulging of eyes, large ears, short and stumpy legs, short tail, round distended abdomen, hairless body, pot belly, and excessive lipomatous mass in the abdominal cavity. It was supposed to be a simple autosomal recessive defect with some alterations. In MIRB we observed a craniofacial malformation characterized by underdeveloped incisive, maxillary and nasal bones with a consequent tongue prolapse, and lower jaw deformities (Figure 2) that, according to literature [58,75] can be classified as another case of “Bull Dog” calf. The absence of conditions like maternal infections and teratogenic factors exposure assume a genetic cause behind the malformation of this calf. 

## 5. Disorders of Sexual Development 

Disorders of sexual development (DSDs) are congenital conditions in which chromosomal, gonadal, or anatomical sex development is atypical [77]. Sexual development is based on three essential steps: (1) the establishment of chromosomal sex which occurs at the time of fertilization; (2) the determination of gonadal sex; and (3) the development of phenotypic sex; the last two steps are under genetic control.

The abnormalities of chromosomal sex are linked to abnormalities in sex chromosome number or structure. In the river buffalo, cases of X-monosomy (Turner’s syndrome) [78,79], X-trisomy [80,81], and XXY chromosome complemented with translocation between the two X chromosomes [82] have been reported. It has been estimated that about 20% of MIRB females with reproductive problems and examined with cytogenetic analyses are carriers of sex chromosome abnormalities [83]. In domestic animals the X-monosomy is a rare congenital condition due to the absence of one X-chromosome and has been reported in two Murrah and two MIRB buffaloes [8,79]. Generally the carrier is sterile with serious modifications of the reproductive apparatus, such as an abnormal cervix and uterus, or the absence of gonads. The X-trisomy has been found in two river buffaloes, one Murrah [80] and one MIRB [7]. Both animals were sterile because of abnormalities of internal genitalia (absent gonads). For the first time Patel et al. [82] reported a case of a Mehsana buffalo bull with an XXY chromosome complement and with a translocation between the two X chromosomes. The bull was phenotypically normal with regular reproductive behavior but sterile, due to azoospermia.

The abnormalities of gonadal sex are related to mutation, deletion, or translocation of small parts of chromosome containing one or more genes which regulate the testis and ovarian developing pathways, like *SRY*, *SOX9*, *RSPO1*, and *WNT*. These abnormalities are defined as sex reversal disorders and classified according to the chromosomal complement detected in the individual (male or female) and the presence/absence of *SRY* gene [84]. Therefore, there are four conditions: XX male SRY-negative, XX male SRY-positive, XY female SRY-negative, and XY female SRY-positive [85]. 

In domestic animals sex reversal disorders are rare and described in horses [84,85,86], cattle [87], pigs [88], dogs [89], and cats [90]. Up to now, in river buffalo only the XY female condition in two MIRBs with female external genitalia and some phenotypic male traits [7] has been reported. Both animals were sterile due to gonadal dysgenesis; in particular, one was SRY positive and lacking internal sex ducts; and the other one (with an unknown *SRY* condition) showed Müllerian duct atrophy. 

The abnormalities of phenotypic sex are related to developmental alterations of internal and external genitalia, which is strictly dependent on secretions from testis in males. In carriers, defined pseudohermaphrodites, the chromosomal and gonadal sex are in agreement, but the internal or external genitalia are ambiguous. The causes of this condition are often unknown because multiple genes are involved in the differentiation of genital apparatus and their roles are still not clear. In dogs, the iatrogenic administration of androgen or progesterone to the dam during gestation could induce masculinization of female fetuses (female pseudohermaphrodites) [89]. In Persistent Müllerian Duct Syndrome (PMDS), a type of canine male pseudohermaphroditism, a mutation of the *MISRII* gene, causes the failure of the regression of Müllerian ducts in males during embryo development [88].

**Figure 3:**
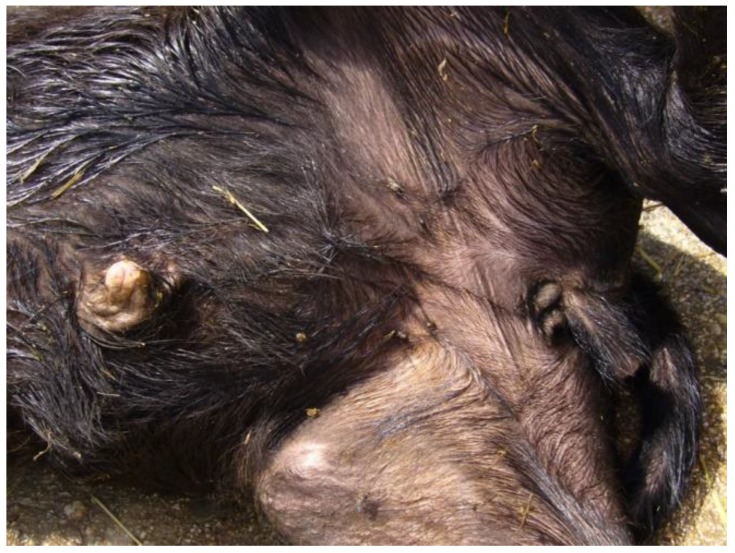
A MIRB male calf with abdominal cryptorchidism and penis craniocaudally directed.

Another disorder of sexual development is cryptorchidism, a condition in which one or both testicles fail to descend into the scrotum and it is an abnormality in the development of phenotypic sex without sexual ambiguity. In addition to environmental factors, like endocrine disruptors, cryptorchidism is at least in part determined by genetic causes and, until now, many candidate genes have been identified [91]. It is very common in livestock and pets, while, in river buffalo, only one case of cryptorchidism has been described in a Pandharpuri calf associated to umbilical hernia. In the affected calf both testicles were located ectopically under the skin of the ventral abdomen alongside the penis [39]. In MIRB, we have examined a male calf with bilateral abdominal cryptorchidism and a penis craniocaudally directed; the calf was born healthy and at the end of gestation (Figure 3). No karyotype abnormalities were found in this calf and gene analyses are still ongoing. 

## 6. Other Malformations

### Meccanobullosus Acantholytic Dermatosis

This condition is very rare and characterized by a reddish skin that is easy to lacerate, with detachment of the epidermic tissue. At birth the animal is normal, but at 2–3 days of age tufts of hair fall out and the skin starts to lacerate; moreover, injuries to the corneal layer of the hoof may occur. With respect to river buffalo, it has been reported in four Murrah calves transmitted with an autosomal recessive gene and due to the reduced functionality of cadherins [5].

## 7. Conclusions

Congenital malformations due to genetic factors represent a significant limit for selection plans and for animal welfare when the underlying gene mutations are unknown and carrier identification is not possible until the mutation is so widespread as to be phenotypically evident. 

This is typically due to an increase of the inbreeding rate and the spreading of negative alleles responsible for genetic diseases. To avoid this problem, it is necessary not only to set up breeding plans that avoid the increase in inbreeding, but also to know genetic conditions underlying the diseases and exclude carriers from mating. The completion of buffalo genome sequencing would represent another useful tool to discover those conditions, but in order that these mutations are discovered, a good recording system is necessary and breeders and institutions must be sensitized to the importance of reporting and examining all new cases of malformations, sampling all cases in which genetic causes are assumed. According to the literature and our personal experience, river buffalo have a great variety of malformations due to genetic causes, and TH and omphalocele are the most frequent. However, several cases are still not reported, leading to an underestimation of the real weight of genetic diseases in this species. This theory is confirmed by the fact that, despite 95% of the world buffalo population being bred in Asia, the number of malformations reported in the literature is extremely small, in contrast to what is observed in countries, such as Brazil and Italy, where only 2% of the buffalo world population is bred. Moreover, except for DSDs for which genetic causes have been identified and diagnostic tests are available, for all other malformations, the genetic causes are unknown. In conclusion this report represents a reference point to focus future studies on the most important malformations that affect river buffalo and that compromise the results of genetic improvement plans and the welfare of the animals.

## Figures and Tables

**Table 1:** Case number and types of congenital malformations reported in literature in different river buffalo breeds.

Congenital Malformations	Breed	Cases (n)	References
**Musculoskeletal defects**			
Transverse Hemimelia	MIRB	10	[6]
	MIRB	13	[10]
	Indian Meshana	1	[12]
	MIRB	91	Present study
	unknown	1	[13]
Artrogryphosis	MIRB	1	[9]
	Murrah	6	[20]
	Murrah	1	[21]
**Other musculoskeletal defects**			
Perosomus elumbis	Nili Ravi	1	[22]
*Campylorrhacchis contorta*	unknown	1	[26]
Myotonia congenita	Murrah	29	[4]
Megaesophagus	Murrah	9	[5]
Other limb malformations	MIRB	2	Present study
Omphalocele	MIRB	72	[9]
	Pandharpuri	1	[39]
Schistosoma Reflexum	MIRB	2	[9]
	Murrah	1	[44]
**Gastrointestinal defects**			
Atresia ani	MIRB	2	[9]
**Craniofacial abnormality**			
Hydrocephalus	MIRB	3	[9]
	Murrah	7	[4]
	Surti	1	[58]
	–	1	[59]
Polycephaly	MIRB	1	Present study
	–	1	[68]
	–	1	[60]
	–	1	[67]
	Nili Ravi	1	[69]
Other Craniofacial malformations	Murrah	2	[75,76]
	Surti	1	[58]
	MIRB	1	Present study
**Disorders of sexual development**			
X-monosomy	MIRB	2	[7]
X-Trisomy	MIRB	1	[7]
Sex reversal	MIRB	2	[7]
Cryptorchidism	MIRB	1	Present study
**Other malformations**			
Meccanobullosus acantholytic dermatosis	Murrah	4	[5]

**Table 2:** Phenotypic trait of transverse hemimelia (TH) cases and their frequency in Mediterranean Italian River Buffalo (MIRB) of Italian breeding farms (data from the Department of Veterinary Medicine and Animal Productions, University Federico II, Naples).

Malformed Limb	Anatomic Structures Involved	Frequency (%)
Left hind limb	Proximal epiphysis tibia	6
	Distal epiphysis tibia	2
	Tarsus	10
	Proximal epiphysis metatarsus	8
	Distal epiphysis metatarsus	10
	Proximal epiphysis of first phalanx	8
Right hind limb	Proximal epiphysis tibia	12
	Tarsus	2
	Proximal epiphysis metatarsus	6
	Distal epiphysis metatarsus	6
Both hind limbs	r. distal epiphysis tibia l. proximal epiphysis metatarsus	6
	r. distal epiphysis tibia l. distal epiphysis metatarsus	2
	r. metatarsus l. metatarsus	8
	r. second tarsus bones l. second phalanx	2
	r. knee l. twisted	2
Both hind limbs and one forelimb	r. hind limb second tarsus bones l. hind limb diaphysis tibia l. forelimb loss of the third phalanx	2
	r. hind limb second tarsus bones l. hind limb proximal epiphysis metatarsus r. forelimb hypoplasia	2
	Amelia of both hind limbs r. forelimb longitudinal ectromelia of metacarpus	2
All limbs involved		4

r. = right; l. = left.
